# Genome-Wide Analysis of Abnormal H3K9 Acetylation in Cloned Mice

**DOI:** 10.1371/journal.pone.0001905

**Published:** 2008-04-09

**Authors:** Takahiro Suzuki, Shinji Kondo, Teruhiko Wakayama, Paul E. Cizdziel, Yoshihide Hayashizaki

**Affiliations:** 1 Genome Exploration Research Group, RIKEN Genomic Sciences Center (GSC), RIKEN Yokohama Institute, Yokohama, Kanagawa, Japan; 2 Division of Genomic Information Resources, Supramolecular Biology, International Graduate School of Arts and Sciences, Yokohama City University, Yokohama, Japan; 3 Laboratory for Genomic Reprogramming, Center for Developmental Biology, RIKEN Kobe, Kobe, Hyogo, Japan; 4 Genome Science Laboratory, Discovery and Research Institute, RIKEN Wako Main Campus, Wako, Saitama, Japan; 5 Functional RNA Research Program, RIKEN Frontier Research System, Wako, Saitama, Japan; Fred Hutchinson Cancer Research Center, United States of America

## Abstract

Somatic nuclear transfer is a cloning technique that shows great promise in the application to regenerative medicine. Although cloned animals are genetically identical to their donor counterparts, abnormalities in phenotype and gene expression are frequently observed. One hypothesis is that the cause of these abnormalities is due to epigenetic aberration. In this report, we focused our analysis on the acetylation of histone H3 at lysine9 (H3K9Ac). Through the use of whole genome tiling arrays and quantitative PCR, we examined this epigenetic event and directly compared and assessed the differences between a cloned mouse (C1) and its parental nuclear donor (D1) counterpart. We identified 4720 regions of chromosomal DNA that showed notable differences in H3K9Ac and report here many genes identified in these hyper- and hypo-acetylated regions. Analysis of a second clone (C2) and its parental donor counterpart (D2) for H3K9Ac showed a high degree of similarity to the C1/D1 pair. This conservation of aberrant acetylation is suggestive of a reproducible epigenetic phenomenon that may lead to the frequent abnormalities observed in cloned mice, such as obesity. Furthermore, we demonstrated Crp which was identified as a hyper-acetylated gene in this study is related to the body mass, suggesting that Crp is a possible candidate of a cause for the abnormal obesity in cloned mice. In this, one of the first reports describing genome-wide epigenetic abberation between parental and nuclear transfer-cloned mammals, we propose that aberrant acetylation of histones (H3K9Ac) flanking promoter regions highly correlates with gene-expression and may itself be an epigenetic change that accounts for variable expression patterns observed in cloned animals.

## Introduction

Because success in cloning techniques using somatic nuclear transfer (NT) has led to the generation of genetically identical animals, the technique is now being developed for regenerative medicine. But despite substantial improvements in mouse NT cloning during recent years [1]–[7], the cloning efficiency remains low. The best success rate is only approximately7 percent [4]. After birth, cloned offspring frequently exhibit a variety of abnormal phenotypes, some of which commonly include an increased body weight (large fetus syndrome) [8], placental overgrowth [9], immuno-system anomalies, and a short life span [10]. Since the environment for fetal development largely influences health and disease of post-natal life (so called “barker's hypothesis”) [11], [12], the abnormal phenotypes may be derived from aberrations in nascent stage. Therefore, these low efficiency and abnormalities may be a consequence of physical damages from a piezo-assisted NT[13], epigenetic changes caused by *in vitro* culture prior to implantation, and failure or incomplete nuclear-reprogramming. Furthermore, different uterine environment from the donor may contribute to the abnormalities as well. In many labs, general attempts to identify abnormalities at a molecular level revealed atypical profiles of gene expression compared to naturally conceived animals. These abnormal gene expression patterns were observed not only in early development, such as in embryos but also in placentas and normally birthed cloned mice [14]–[17]. Kohoda *et al*. have reported that cloned mice offspring show an over-diversity of gene expression compared to normal mice, but common abnormalities are surprisingly few. They also showed that some of these genes map in a linked cluster and are commonly changed in the same manner. Taken together, this evidence suggest that although there are many abnormalities caused by random components, cloned mice indeed exhibit common abnormalities that result from non-random components.

DNA methylation and histone modifications are known to be important epigenetic events that control cellular morphology and function [18], [19]. There is now a growing body of evidence suggesting that proper histone acetylation may be a critical element in successful NT cloning. Treatment with trichostatin A (TSA), a histone deacetylase inhibitor, after somatic nuclear transfer significantly improves the mouse cloning not only in the generation rate but also in reducing the abnormal phenotypes, with the exception of placenta overgrowth [4], and enables mouse cloning of an outbred strain[6]Furthermore, the developmental potential of clones correlate with the histone acetylation levels in rabbits [20] and primates[21]. These reports clearly indicate that histone acetylation is a key element in animal cloning but the mechanism that links histone acetylation to phenotypes remains undetermined.

Acetylation of histone H3 at Lysine 9 (H3K9Ac), one of the most well known epigenetic markers, is enriched in the promoter region of activated genes [22]–[25]. The level of H3K9Ac in the promoter strongly correlates to the gene expression. High density oligo tiling arrays which cover entire genomes have enabled the genome wide analysis not only of transcriptome by RNA mapping [26]–[29] but also of protein-DNA interactions or histone modifications by a combination with chromatin immunoprecipitation (ChIP) [22]–[24], [30]. Hence, we have obtained genome-wide data of H3K9Ac by using commercially available whole-genome tiling arrays.

Application to a regenerative medicine, a comparison of clones to their donor counterparts may provide more valuable insights than comparing clones and normal animals. However, nearly all studies of cloned mice so far, have focused on this latter comparison. In the gene expression analysis, individual cloned mice show diverse results, suggesting that combined data from multiple cloned mice will not accurately represent abnormalities or differences of significance [16]. Therefore, our approach focused on investigating differences between a single cloned mouse and its parental (donor) counterpart. In this report we characterized one key epigenetic abnormality at the genome-wide level, analyzing the level of H3K9Ac in the liver of the clone and that of its parental (donor) counterpart by using whole-genome tiling arrays. We identified genomic regions that showed a notable difference of H3K9Ac between the mouse clone and donor and identified genes in those regions whose promoters were either significantly hypo-acetylated or hyper-acetylated. We also have shown that abnormal gene expression in a cloned mouse often correlates with abnormal H3K9Ac at their promoters. These findings suggest that, among epigenetic factors, H3K9Ac may be one of the major causative elements of abnormalities that need to be addressed for improving success rates in animal cloning or perhaps applications in regenerative medicine.

## Results

### H3K9 acetylation profiles of cloned mouse and its donor mouse by *ChIP on chip*

To identify the H3K9Ac enriched sites, we performed ChIP followed by tiling array analysis (*ChIP on chip*) using the Affymetrix mouse tiling 1.0 array, which spans the entire mouse genome at a 30 bp resolution with 47 million 25-mer probes (excluding repetitive sequence elements). Fixed chromatin preparations from cloned mouse (C1) and donor mouse (D1) livers were immunoprecipitated with an antibody directed against the H3K9Ac. The immunoprecipitated DNA (treatment sample) and the control DNA from a whole cell extract (WCE) were amplified by the Ligation-Mediated PCR (LM-PCR) method and hybridized to the tiling arrays as previously described [23]. As a measure of the H3K9Ac enrichment, a P-value (P) was computed at each probe position by assessing the excess of signal intensities detected by probes located within±200 bp in the treatment relative to the control sample by wilcoxon rank sum test [30]. Using P≤10^−4^ as a significant cut-off [22], [23], we have identified 30902 and 32757 H3K9Ac enriched sites in the C1 and the D1 livers, respectively. Among them, 9584 sites (31%) and 10201 sites (31%) were located in the promoter regions which are defined as being±1 kb from transcription start site (TSS). The TSSs were determined by aligning 470,133 RIKEN 5′ ESTs[31] to the mouse genome, by using BLAT[32] and SIM4[33]. These results clearly indicated that the H3K9Ac enriched sites were biased toward the promoter regions, which is consistent with previous studies performed on human cell lines [22]–[25], [34], and suggest this regional bias and functions of H3K9Ac are conserved among higher eukaryotes.

### Validation for tiling array data

**Figure 1:**
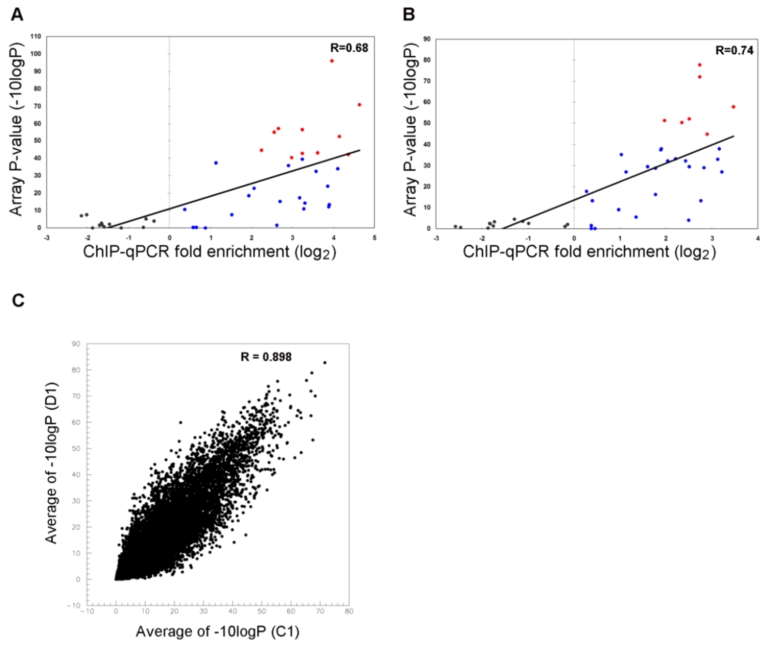
* ChIP on chip* data Analysis. (A, B) Relative enrichment measured by ChIP-qPCR for 43 sites in C1 (A) and D1 (B) and the average of −10logP of each of 43 regions were plotted as a scatter plot. Black points represent the un-enriched amplicons, blue points represent the enriched amplicons of qPCR data where −10logP<40 for *ChIP on chip*, red points represent the enriched amplicons of qPCR data where −10logP≥40 for *ChIP on chip*. (C) Averages of −10logP at each promoter (±1 kb of TSS) were plotted as a scatter plot. Vertical axis represents D1 liver and the horizontal axis represents C1 liver. Total promoter sites used in this analysis included 16203 sites. Spearman correlation coefficient between C1 and D1 was 0.898.

Although *ChIP on chip* data is a useful mass screening tool, the technology is less reliable for quantitation of DNA than many other methods. Hence we selected quantitative PCR (qPCR) as a validation tool and picked 43 genomic sites to test. No consideration was given to the P-values of the selected sites but rather they were chosen for their general dispersion across the genome (Table S1). Of these 43 sites, 23 were in the gene promoters and 20 sites were located among intergenic sequences. Using identical immuno-precipitation techniques, we carried out ChIP followed by qPCR (ChIP-qPCR) on the 43 sites [35]. qPCR was achieved by real-time PCR and the enrichment of the ChIP DNA as multiples of the WCE were computed for each site. The quantitative values for enrichment as determined by ChIP-qPCR were plotted against P-values measured by *ChIP on chip* (Figure 1A, B). Sites displaying little to no enrichment [log_2_] measured by ChIP-qPCR coordinately showed very low significance P-values as measured by *ChIP on chip* (values of 10 and below in -10logP). In the sites showing 2-fold enrichment and above (≥log_2_1), increasing enrichment clearly correlates with an increasing significance level of P-value. Out of 18 sites, Seventeen (17) sites which were detected as P≤10^−4^ yielded an enrichment greater than 4-fold in ChIP-qPCR (11/11 in C1 liver, 6/7 in D1 liver). Based on this, we conclude that the cut-off of P≤10^−4^ is reliable to identify the H3K9Ac enriched sites with little false positives and high certainty. Furthermore, the total Pearson correlation coefficients were 0.68 and 0.74 in C1 and D1 liver samples, respectively. In addition, from the data, we can interpret that amplification of ChIP DNA by LM-PCR prior to hybridization to the tiling array did not introduce a significant bias into the *ChIP on chip* results.

### Promoter H3K9 acetylation differences between Cloned and Donor mouse

Promoter regions are a major target for epigenetic studies to explain gene expression level changes between cloned mice and their parental counterparts. A comparison of the H3K9Ac *ChIP on chip* tiling array P-values from over 16,000 promoter region data points between C1 and D1 livers samples showed a high degree of similarity, with a Spearman correlation coefficient of 0.898 (Figure 1C). However, to specifically identify and focus on promoter regions where significant differences exist in H3K9Ac levels between mouse clones and donors, signal intensities from *ChIP on chip* tiling array data in the C1 sample relative to the D1 sample and *vice versa* were evaluated by the Wilcoxon rank sum test [30]. In the cloned mouse (C1), 2550 hyper-acetylated sites and 2170 hypo-acetylated sites were identified with a P≤10^−4^. Of these sites, 113 and 147 sites respectively, were located in gene promoter regions. These results establish that abnormal H3K9Ac differences between a clone and a parental donor is not significantly biased toward promoter regions, but rather scattered across the chromosomal DNA.

In that changes in gene expression are ultimately responsible for abnormalities in clones, we next focused our analysis on the regions identified that mapped to promoters and were either hyper- or hypo-acetylated at H3K9. To confirm abnormal H3K9Ac of the promoters, we performed ChIP-qPCR using primers designed by Primer3 software from sequence information of the regions that were statistically significant at a P≤10^−4^ or less. From 113 hyper-acetylated and 147 hypo-acetylated site possibilities, only 74 and 61 primer sets could be designed for validation due to sequence constraints (Table S2). As before, ChIP DNA was amplified by LM-PCR method followed by real-time PCR measurement. The relative difference in the level of H3K9Ac between the samples was computed as the change in the C1 relative to the D1 sample as log_2_ (C1/D1). Cut-off values for this comparison were relaxed relative to the previous ChIP-qPCR analysis because the reference material was another ChIP sample (not WCE) and as a consequence, the results displayed more variability. In addition, since the difference of H3K9Ac was relatively insignificant between C1 and D1 (Figure 1C), cut-off value should be looser to identify imperceptible differences. Furthermore, stringency did not need to be high at this stage of selection since a first screening was already performed by tiling array data. Thus, we needed here sensitivity rather than specificity. A significant increase (>0.5 [log_2_ (C1/D1)]) of an H3K9Ac level in the C1 sample was detected on 26 hyper-acetylated sites (Table 1), and a significant decrease (<−0.5 [log_2_ (C1/D1)]) was detected on 22 hypo-acetylated sites (Table 2).

**Table 1:** Hyper-acetylated Genes of Mouse Clone (C1)

		Change in acetylation	
Accession No.	Gene name	Percent (%)	log2
NM_145367.3	thioredoxin domain containing 5 (Txndc5)	187.15	0.90
NM_021462.2	MAP kinase-interacting serine/threonine kinase 2 (Mknk2)	180.98	0.86
NM_007886.1	dystrobrevin, beta (Dtnb)	177.55	0.83
NM_007768.2	C-reactive protein, pentraxin-related (Crp)	146.00	0.55
NM_030021.1	RIKEN cDNA D730039F16 gene (D730039F16Rik)	164.60	0.72
NM_172476.2	transmembrane channel-like gene family 7 (Tmc7)	222.29	1.15
NM_053183.1	DEAD (Asp-Glu-Ala-Asp) box polypeptide 50 (Ddx50)	203.71	1.03
NM_024440.1	Der1-like domain family, member 3 (Derl3)	681.68	2.77
NM_010301.2	guanine nucleotide binding protein, alpha 11 (Gna11)	221.87	1.15
NM_177152.4	leucine-rich repeats and immunoglobulin-like domains 3 (Lrig3)	304.04	1.60
NM_177614.2	amplified in osteosarcoma (Os9)	141.57	0.50
NM_011814.2	fragile×mental retardation, autosomal homolog 2 (Fxr2),	160.83	0.69
NM_153103.1	kinesin family member 1C (Kif1c)	174.76	0.81
NM_023113.3	aspartoacylase (aminoacylase) 2 (Aspa)	145.37	0.54
NM_153804.3	pleckstrin homology domain containing, family G (with RhoGef domain) member 3 (Plekhg3)	149.89	0.58
NM_144836.2	solute carrier family 17 (sodium phosphate), member 2 (Slc17a2)	200.88	1.01
NM_183146.2	RIKEN cDNA A530054K11 gene (A530054K11Rik)	153.97	0.62
NM_001033988.1	nuclear receptor coactivator 4 (Ncoa4), transcript variant 2	160.09	0.68
NM_007447.2	angiogenin, ribonuclease A family, member 1 (Ang1)	145.98	0.55
NM_020271.2	pyridoxal (pyridoxine, vitamin B6) phosphatase (Pdxp)	389.88	1.96
NM_144942.1	cysteine sulfinic acid decarboxylase (Csad)	339.34	1.76
NM_133666.1	NADH dehydrogenase (ubiquinone) flavoprotein 1(Ndufv1)	151.52	0.60
NM_011697.1	vascular endothelial growth factor B (Vegfb)	158.46	0.66
NM_144873.1	ubiquitin-like, containing PHD and RING finger domains2 (Uhrf2)	160.35	0.68
NM_138595.1	glycine decarboxylase (Gldc)	214.93	1.10
NM_175507.2	transmembrane protein 20 (Tmem20)	174.02	0.80

The change in acetylation is displayed as both percent change (%) and log (base2) values for the C1 animal relative to D1. Each value represents the average of two replicates ChIP-qPCR assays.

**Table 2:** Hypo-acetylated Genes of Mouse Clone (C1)

		Change in acetylation	
Accession No.	Gene name	Percent (%)	log2
NM_001035531.1	adrenergic receptor kinase, beta 2 (Adrbk2), transcript variant 2	51.44	−0.96
NM_007925.2	elastin (Eln)	35.82	−1.48
NM_177678.4	actin-binding LIM protein 2 (Ablim2)	54.50	−0.88
NM_153501.1	pantothenate kinase 2 (Hallervorden-Spatz syndrome) (Pank2)	54.62	−0.87
NM_178768.3	RIKEN cDNA C230095G01 gene (C230095G01Rik)	11.53	−3.12
NM_172759.1	carboxylesterase 5 (Ces5)	67.77	−0.56
NM_009736.1	Bcl2-associated athanogene 1 (Bag1)	66.58	−0.59
NM_010451.1	homeo box A2 (Hoxa2)	50.63	−0.98
NM_026637.2	RIKEN cDNA A030007L17 gene (A030007L17Rik)	58.92	−0.76
NM_175268.4	RIKEN cDNA A930008G19 gene (A930008G19Rik)	33.46	−1.58
NM_212473.1	RIKEN cDNA A930008G19 gene (A930008G19Rik)	46.71	−1.10
NM_030254.2	tumor suppressor candidate 3 (Tusc3)	61.09	−0.71
NM_030152.2	nucleolar protein 3 (apoptosis repressor with CARD domain) (Nol3)	38.53	−1.38
NM_133792.2	lysophospholipase 3 (Lypla3)	64.77	−0.63
NM_009698.1	adenine phosphoribosyl transferase (Aprt)	60.82	−0.72
NM_178933.2	organic solute transporter beta (Ostb)	41.38	−1.27
NM_175206.2	F-box and leucine-rich repeat protein 22 (Fbxl22)	59.27	−0.75
NM_153145.2	ATP-binding cassette, sub-family A (ABC1), member 8a (Abca8a)	25.92	−1.95
NM_025695.3	structural maintenance of chromosomes 6 (Smc6)	15.98	−2.65
NM_198301.1	cDNA sequence BC052328 (BC052328),	44.39	−1.17
NM_054052.2	UDP-GlcNAc:betaGal beta-1,3-N-acetylglucosaminyltransferase 5 (B3gnt5)	68.42	−0.55
NM_027222.1	RIKEN cDNA 2010001M09 gene (2010001M09Rik)	40.01	−1.32

The change in acetylation is displayed as both percent change (%) and log (base2) values for the C1 animal relative to D1. Each value represents the average of two replicates ChIP-qPCR assays.

### Relationship between abnormalities of H3K9 acetylation and gene expression

**Figure 2:**
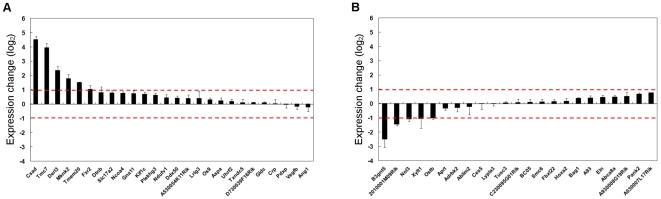
Expression Modulation of Genes with Promoters Hyper- and Hypo-acetylated at H3K9. Hyper-acetylated genes (A) and Hypo-acetylated genes (B) were pre-selected by *ChIP on chip* data and validated by ChIP-qPCR for alterations in H3K9Ac, and then analyzed for changes in gene expression by qRT-PCR (n = 2). Bars are shown as the average of duplicate assay results for changes in expression levels in C1 relative to D1. Standard deviations are shown using error bars. Upper red dotted lines represent 2-fold increase and lower red dotted lines represent 2-fold decrease.

Previous studies have shown that the level of H3K9Ac in promoter regions is strongly correlated with gene expression[23]. Our hypothesis was that hyper- and hypo-acetylation in the promoter region leads to abnormal gene expression in a cloned mouse. To test this hypothesis, the expression levels of the genes whose promoters were hyper- or hypo-acetylated in the C1 and D1 mice were measured by using qRT-PCR. Total RNA was isolated from livers of the C1 and the D1 mice, and reverse-transcribed with random hexamers. Using the cDNA as a template, a real-time PCR measurement was performed using coding region primers (Table S3) for the 26 hyper-acetylated genes (Table 1) and 22 hypo-acetylated genes (Table 2) from the C1 and D1 mice. We then determined the differences in gene expression between the C1 liver and the D1 liver using the 2^−ΔΔCt^ method[36] (Figure 2). We detected 2-fold or greater increase in the level of expression for 6 (23.1%) out of the 26 hyper-acetylated genes, and none showed a 2-fold or greater decrease in gene expression. For the 22 hypo-acetylated genes examined, we found 5 (21.7%) that showed 2-fold or greater decrease in the level of expression and none that showed 2-fold or greater increase in expression levels. These results suggest that hyper- or hypo-acetylation of H3K9Ac may contribute to a part of the abnormal gene expression in the NT cloned mice, but is likely not the only factor involved.

### Conservation of H3K9Ac variation in other mouse clones

**Figure 3:**
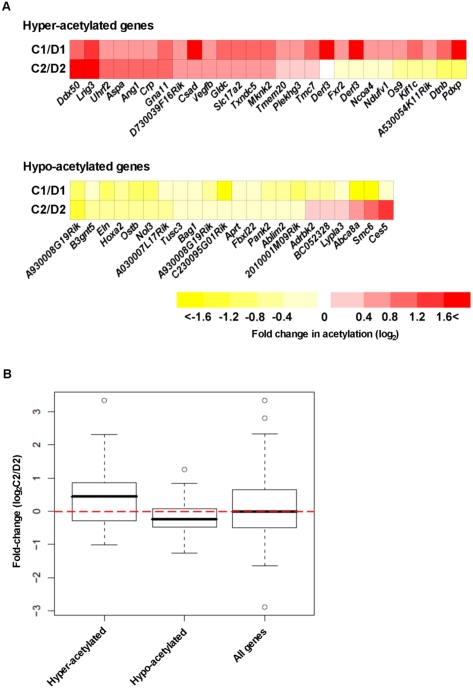
Conservation of H3K9Ac variation in other mouse clones. Acetylation-state changes of each promoter site in the cloned mice are indicated by color, and as a multiple of corresponding donor mice (A). No-change is represented as white, increased acetylation is depicted as intensifying red color, and decreased acetylation is depicted as intensifying yellow color. The value of each change is the average of technical replicates (n = 2). Details of the target genes are reported in Table 1 and Table 2. (B) For each gene, the actual experimental values for the C2/D2 pair is graphically aligned in categories of hyper- or hypo-acetylation containing 26 and 22 genes, respectively, which is a reflection of gene-state determined in the C1/D1 mouse pair. Medians for each data set are indicated by black center lines, upper quartiles are indicated by upper edges of the box, and lower quartiles are indicated by lower edges of the box. Maximum and minimum values are marked as end of lines extending from the boxes. Values which are more than 1.5 times the inter-quartile range from the upper or lower quartile are represented as white circles and are considered outliers and not used in the calculations. The red dotted horizontal line indicates the position of median of all genes (hyper- and hypo-acetylated genes, total 48 genes).

It is important to determine if the same aberrant acetylation events are reproducible and lead to the reproducible abnormal phenotypes observed in cloned mice. To investigate this, we examined another cloned mouse (C2) and donor (D2) pair that was derived using the same cumulus cell NT technique. We repeated the ChIP-qPCR experiment using the same primer sets for 26 hyper- and 22 hypo-acetylated genes (Table S2) on ChIP from the livers of the C2 and D2 mice. The change in acetylation for each gene for the C1/D1 pair and C2/D2 pair (Figure 3A) was statistically analyzed for group significance (Figure 3B). Among the hyper-acetylated sites, the overall tendency of changes in the C2/D2 pair was clearly biased toward the positive compared to all (hyper- and hypo-acetylated) sites, with a P<0.05 (Welch's t-test), indicating statistical significance toward hyper-acetylation (Figure 3B). On the other hand, the overall distribution of the hypo-acetylated sites was biased toward negative change with a P<0.05 (Welch's t-test), indicating statistical significance toward hypo-acetylation (Figure 3B). These results suggest that the abnormalities in H3K9Ac patterns among cloned mice derived from cumulus cells by NT tends to be conserved among cloned individuals, and the abnormalities may be related to common abnormal phenotypes of cloned mice.

## Discussion

The abnormalities of cloned mice have been recognized not only by phenotype [8]–[10] but also partially characterized by gene expression [14]–[17]. The root causes of these abnormalities are thought to be attributed to multiple factors. For instance, a growing body of evidence has emerged to support the general supposition that chromosomal abnormalities are one of the elements for failures in animal cloning [13], [37].Among these factors, epigenetic factors are important elements for the successful animal cloning[3], [4], [6], [20], [21]. However, the epigenetic properties responsible for the aberrant cloned mouse phenotypes are poorly understood. We have chosen to analyze H3K9Ac because much evidence exists relating this epigenetic feature to active gene expression [22]–[25]. Previous studies have focused on normal mice as the reference control [9], [10], [14]–[17], [38], but not specifically the parental NT-donor mouse. A comparison of the clone with its donor, more accurately identifies the variation of H3K9Ac that occurs in cloning and suggests that a large number of genomic sites are involved. We also examined whether the same H3K9Ac alterations existed in two pairs of cumulus cell-derived cloned mouse and parental donor pairs. Evidence showed that the promoter region hyper- and hypo-acetylated sites detected in one cloned mouse tended to be similar and statistically significant in the other examined cloned mouse, as well. However, it is not clear if this conservation of the H3K9Ac alteration pattern among separate clones is an inheritance from the donor cell, or if it results from the aberrant nuclear-reprogramming which is based on common factors. To answer this question, further study is required.

In higher eukaryotes, transcriptional regulation is complex. Many factors and elements influence and directly regulate transcription, such as transcriptional regulatory factors, DNA methylation, and Chromatin structure. Furthermore, these elements work together and clearly influence one other, establishing a concerted functional “network” [39]. Although we previously reported that the degree of H3K9Ac and gene expression showed positive correlation, we also found many examples that are not consistent with this observation [23]. Another group also reported that the histone modifications which are known to be associated with active gene promoter are enriched at both active and inactive promoters [34]. Hence, the cause of abnormal gene expression is not solely a direct consequence of H3K9Ac, but perhaps an indirect consequence or a contributing factor influenced by other elements as well. From our experiments looking at genes with aberrant H3K9Ac, almost one-fourth of the hyper-acetylated genes we examined for expression in clone mouse (C1) livers were up-regulated by more than 2-fold, and almost one-fifth of the hypo-acetylated genes were down regulated when compared to the donor animal (D1) liver. It is possible that H3K9Ac increases the possibility of active transcription, but itself is not sufficient [24], [34]. The correlation between gene expression and H3K9Ac presented in this study is consistent with the possibility. This may explain the phenotypic variability in TSA treated cloned mice. We believe that aberrant H3K9Ac in cloned mice is a critical factor influencing abnormal gene expression, but by simply looking at one tissue (such as liver in this study), or embryonic stage, or animal age, the real overall impact cannot be fully determined. One interesting finding is that H3K9Ac enriched sites are clearly biased toward gene promoters. However, approximately two-thirds of H3K9Ac was also scattered among the chromosomes, which is consistent with previous studies in human [22]–[24], [34]. Many intergenic sites (>2000) showed a significant difference in the H3K9Ac between cloned and donor mice in this study. Hence it is possible that H3K9Ac influences not only to proximal regulatory elements in and around promoters, but also distant regulatory elements, such as enhancers, silencers, insulators, and locus control regions. This can also have a significant impact on the expression of genes located in the vicinity, and is an important subject for future investigation.

**Figure 4:**
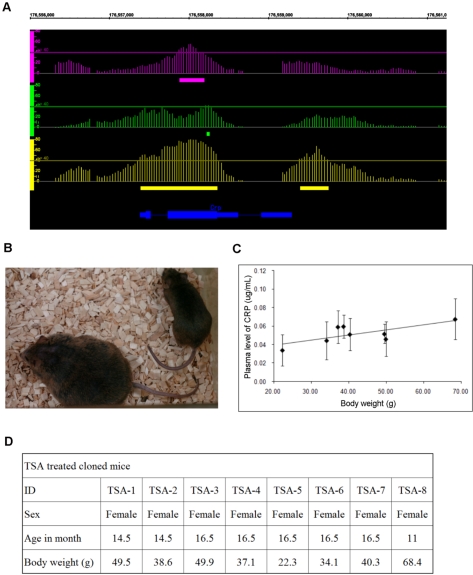
Crp levels in the blood of adult TSA-treated cloned mice of various weights. (A) Result of *ChIP on chip* analysis at the region of the Crp gene. Horizontal axis represents alignment of Chr1 and the vertical axes represent P-value of tiling array intensity data. The scale of the genome viewer is given as coordinate of Chr.1 (bp). Upper tier (magenta) represents direct comparison of C1 ChIP sample vs. D1 ChIP sample, middle tier (green) represents D1 ChIP sample vs. WCE, and lower tier (yarrow) represents C1 ChIP sample vs. WCE. Each vertical line standing on the each tire represents positions of array probes and its −10logP value. Horizontal lines in each tier represent P = 10^−4^ and filled bars being bottom of each tier represent regions which are P<10^−4^ or less, respectively. Blue line represents the exon structure of the Crp gene. (B) Photograph of TSA-treated mice used for Crp plasma protein level measurements by ELISA. Right mouse is the leanest mouse (TSA-5) and left mouse is the most obese mouse (TSA-8). (C) Crp protein concentration in TSA-treated mouse clone serum measured by ELISA. The vertical axis represents the concentration of Crp protein in serum and the horizontal axis represents body weight. The concentration is shown as the average of four technical replicates (n = 4). Pearson correlation coefficient for the test is 0.72. (D) General information of TSA-treated mice. The information shows ID for the mice and corresponding sex, age (month), and body weight.

As a possible phenotype-related H3K9Ac aberration, we noted C-reactive protein (Crp) is a hyper-acetylated gene identified in this study (Figure 4A). Crp is now recognized as a potential predictor of metabolic syndrome [40]. Although there are several disputations [41]–[43], Chen *et al.* reported that Crp induces leptin resistance, inhibiting the binding of leptin to its receptor [44]. In humans and rodents, leptin concentration in the blood shows strong positive correlation with body mass [45], [46], which may be explained by the leptin resistance [47], [48]. Interestingly, one of the common abnormal phenotypes in cloned mice is obesity and the concentration of leptin in the blood is usually more than 10 times higher than that of normal mice [8]. Hence, we speculated that the reason for the obesity in cloned mice is the leptin resistance induced by an excess of the Crp protein. To investigate a relationship between the body mass and the Crp in cloned mice, we measured mice body weight and the Crp protein level in serum by the Enzyme-Linked ImmunoSorbent Assay (ELISA) from eight (8) cloned mice treated with TSA. TSA treatment in mouse cloning partially abrogates the tendency toward obesity, generating cloned mice that have the same background but various body weights (Figure 4B, D). The Crp protein level in serum showed a strong correlation with the weight of these animals (Figure 4C). For this experiment, the Pearson correlation coefficient was 0.72. This result suggests that cloned mice obesity could be related to Crp. However, expression of the Crp mRNA was not significantly changed in the C1 liver compared to D1 liver (Figure 2A). H3K9 hyper-acetylation may be not sufficient to increase the active transcription [24], [34]. Hence the abnormal Crp expression may occur at another age and/or in another tissue in the obese cloned animal and is a topic that warrants further investigation. In a recent paper, Senda et al. report that abnormalities of clones fade with aging[49]. The obesity actually appears 8 weeks after birth[8]. Considering their result and our data, it is plausible to assume that some abnormalities can escape the fading.

To our knowledge, this is the first report describing genome-wide epigenetic aberration between donor and cloned animals. We propose that epigenetic abnormalities, such as those in H3K9Ac lead to abnormal gene expression, and that abnormal gene expression results in abnormal phenotypes. Hence, controlling the epigenetic landscape of a cell or by selecting a proper donor cell type possessing epigenetic information suitable to cloning we may be able to improve cloning success rates and the health of cloned animals.

## Materials and Methods

### Production of Cloned mice

BDF1 (C57BL/6×DBA/2) oocytes and fresh isolated BD129F1 (BDF1×129/Sv) cumulus cells were collected and used for the production of cloned mice as previously described [50]. A group of oocytes was transferred to a droplet of HEPES-CZB [51]containing 5 µg/ml cytochalasin B, then metaphase II chromosome-spindle complexes were removed from the oocytes by an enucleation pipette. The donor cumulus cells were drawn in and out of the injection pipette until the cell membrane was broken and nuclei were injected one by one into an enucleated oocyte. After nuclear transfer, the reconstructed oocytes were activated by 10 mM SrCl_2_ in Ca^2+^-free CZB medium in the presence of 5 g/mL cytochalasin B, and cultured for 24 h in CZB medium. When cloned embryos were developed to the 2-cell stage, it was transferred to the oviduct of foster mothers (ICR strain) which had been mated with vasectomized ICR males 1 day previously. All recipient females were euthanized at 19.5 dpc (days post coitum) and pups raised by lactating ICR foster mothers. All animals (obtained from SLC, Shizuoka, Japan, or bred in Riken) were maintained in accordance with the Animal Experiment Handbook at Riken Center of Developmental Biology.

### Chromatin immunoprecipitation

Livers extracted from cloned and donor mice at the age of 2.5 month-old were homogenized using the BioMasher™ extraction device (Nippi Corp.). ChIP experiments using an anti-acetylated K9 histone H3 antibody (catalog no. 07-352, Upstate Bio Corp.) were performed as described [23] with some modification. Briefly, the homogenized samples were fixed with 1% formaldehyde, resuspended in TE buffer (pH8.0), and fragmented to a size range of 200 to 1000 bases using a Sonifier® S-450 (Branson Crop.) with a cup horn. The fragmented chromatin was pre-cleared with protein G sepharose after adding 100 mM NaCl and 0.4% NP-40. After removal of a control aliquot (whole cell extract, WCE), pre-cleared lysates were incubated at 4°C overnight adding the anti-acetylated K9 histone H3 antibody. The immune complexes were precipitated with protein G Dynabeads® (Invitrogen Dynal Corp.), washed and eluted from beads into elution buffer (100 mM NaHCO_3_, 1% SDS). The WCE aliquot and the eluted immune complexes were reverse cross-linked at 65°C for 3.5h and treated with RNase and Proteinase K. The DNA from WCE and the ChIP DNA was extracted using phenol/chloroform method and ethanol precipitation.

### Ligation Mediated-PCR (LM-PCR) and array hybridization

LM-PCR and array hybridization were performed as previously described [23]. Briefly, the blunted ChIP DNAs and WCE DNAs were ligated with the annealed oligonucleotides, amplified by using Blend *Taq™*-Plus (Cosmo Bio Corp.). The amplified DNA was purified, fragmented with DNase I, and end-labeled with biotin-ddATP by using terminal deoxytransferase. The labeled fragments were hybridized to the GeneChip® Mouse Tiling 1.0 Array Set (Affymetrix Corp.). The arrays were washed, and scanned using the Affymetrix GeneChip® System. Each sample was hybridized and tested as duplicate samples.

### Level of enrichment for acetylation of lysine 9 of histone H3 in the whole mouse genome

The hybridization intensities (background-subtracted intensity, PM–MM where PM and MM indicate intensities detected by a 25-mer perfectly matching and another one-base-mismatching to genome, respectively.) of the probes were measured in two technical replicates for each of the treatment and control samples, and were mapped to the genome using exact 25-mer matching to mouse genome (NCBI version 32). Raw array data were quantile normalized within treatment/control replicate groups and scaled to a median feature intensity of 500. A shift of the intensities in the treatment relative to control data in a 400 bp window centered at each probe was evaluated by a Wilcoxon Rank Sum test, which assigned a P-value to the probe position [22], [23]. We used the Affymetrix software, TAS (http://www.affymetrix.com/support/developer/downloads/TilingArrayTools) for the P-value calculation. All tiling array data including raw signal intensity and calculated P-value are available in Center for Information Biology gene EXpression database (CIBEX) (http://cibex.nig.ac.jp/) accession number CBX38.

### Profile of acetylation at lysine 9 of histone H3 in promoter region of protein-coding genes in donor and cloned mice

The profile of H3K9Ac enrichment in the promoter region (±1 kb of TSS) of protein-coding genes was compared between the donor and cloned mice. Using the 5′ ends of mRNA sequences (18,707 transcripts in 16,720 loci downloaded on Jan. 19, 2006 from RefSeq database: ftp://ftp.ncbi.nih.gov/refseq) aligned to genome as TSS, we attempted to measure the magnitude of H3K9Ac enrichment in the promoter region. The alignment was carried out by using BLAT[32] and SIM4[33]. For the comparison of the H3K9Ac enrichment between the donor and cloned mice, we averaged −10 log P detected by the probes located in the promoter region, and computed correlation coefficient (Spearman).

### Real-time PCR

PCR primers for evaluating ChIP assays (Table S1) were designed to amplify 70–150 base pair fragments from the genomic regions indicated for validation of *ChIP on chip* data, and for identification of the H3K9 hyper- or hypo-acetylated genes (Table S2). PCR amplification was performed on an ABI PRISM® 7500 Sequence Detection System (Applied Biosystems, Inc.). For amplification, SYBR Premix Ex *Taq™* (Takara Corp.) was used as instructed in the manual. The PCR conditions were an initial step of 10 sec at 95°C, followed by 40 cycles of 3 sec at 95°C and 20 sec at 62.5°C. To generate a standard curve for each amplicon, Ct values of serially diluted WCE DNA which extracted in ChIP experiment (5 ng, 2.5 ng, 1.25 ng, 0.625 ng, and 0.3125 ng) were determined and plotted against the amount of WCE DNA template. ChIPed DNA was measured by real-time PCR in duplicates and the median of the average Ct values of all the amplicons in each sample was scaled to 22. The amount of an amplicon was estimated from the standard curve. Enrichment, (fold change or relative enrichment) was determined as multiple of the amount for each amplicon.

### Quantitative Reverse Transcription-Polymerase Chain Reaction (qRT-PCR)

Total RNA was extracted from the livers of the D1 and C1 mice by the acid phenol–guanidinium thiocyanate–chloroform method [52]. Reverse transcription of total RNA was achieved with PrimeScript™ Reverse Transcriptase (Takara Corp.) and random hexamers in accordance with the manufacturer's protocol. Glyceraldehyde-3-phosphate dehydrogenase (GAPDH) mRNA which yielded a amplicon of 76 bp was used as a control (primers 5′-TGTGTCCGTCGTGGATCTGA-3′ and 5′-CCTGCTTCACCACCTTCTTGA-3′) for data normalization. The PCR primers used for each gene in this analysis are given (Table S3). PCR amplification using the real-time PCR was performed as described above. Changes of gene expression were determined using the 2^−ΔΔCt^ method [36].

### Enzyme-Linked ImmunoSorbent Assay (ELISA)

Blood was taken from the eyeground vein of eight TSA-treated cloned mice. After blood clotting, the serum from each sample was recovered as a supernatant by centrifugation. The C-Reactive Protein (CRP) ELISA (High-Sensitivity), Mouse kit (cat. #KT-095, Kamiya Biomedical Company) was used to measure the Crp levels in the serums in accordance with the manufacturer's protocol.

## Supporting Information

Table S1 ChIP-qPCR Primers used for validation of the tiling array data.
(0.02 MB XLS)

Table S2 Hyper- or hypo-acetylated sites (start and end position on the mouse genome NCBI version 32) and primers used for ChIP-qPCR.
(0.05 MB XLS)

Table S3 Hyper- or hypo-acetylated genes and corresponding coding region primers used for qRT-PCR.
(0.02 MB XLS)
